# Complex Mutation Pattern of Omicron BA.2: Evading Antibodies without Losing Receptor Interactions

**DOI:** 10.3390/ijms23105534

**Published:** 2022-05-16

**Authors:** Saathvik R. Kannan, Austin N. Spratt, Kalicharan Sharma, Ramesh Goyal, Anders Sönnerborg, Subbu Apparsundaram, Christian L. Lorson, Siddappa N. Byrareddy, Kamal Singh

**Affiliations:** 1 Bond Life Sciences Center, University of Missouri, Columbia, MO 65211, USA; skannan@missouri.edu (S.R.K.); ans3mm@missouri.edu (A.N.S.); lorsonc@missouri.edu (C.L.L.); 2 Department of Pharmaceutical Chemistry, Delhi Pharmaceutical Sciences and Research University, New Delhi 110017, India; sharmakcpt@gmail.com (K.S.); goyalrk@gmail.com (R.G.); subbu.apparsundaram@gmail.com (S.A.); 3 Division of Clinical Microbiology, Department of Laboratory Medicine, Karolinska Institute, 14186 Stockholm, Sweden; anders.sonnerborg@ki.se; 4 Department of Veterinary Pathobiology, University of Missouri, Columbia, MO 65211, USA; 5 Department of Pharmacology and Experimental Neuroscience, University of Nebraska Medical Center, Omaha, NE 68198, USA; 6 Department of Genetics, Cell Biology, and Anatomy, University of Nebraska Medical Center, Omaha, NE 68198, USA; 7 Department of Biochemistry and Molecular Biology, University of Nebraska Medical Center, Omaha, NE 68198, USA

**Keywords:** COVID-19, SARS-CoV-2, viruses, Omicron BA.1, BA.2, Delta

## Abstract

BA.2, a sublineage of Omicron BA.1, is now prominent in many parts of the world. Early reports have indicated that BA.2 is more infectious than BA.1. To gain insight into BA.2 mutation profile and the resulting impact of mutations on interactions with receptor and/or monoclonal antibodies, we analyzed available sequences, structures of Spike/receptor and Spike/antibody complexes, and conducted molecular dynamics simulations. The results showed that BA.2 had 50 high-prevalent mutations, compared to 48 in BA.1. Additionally, 17 BA.1 mutations were not present in BA.2. Instead, BA.2 had 19 unique mutations and a signature Delta variant mutation (G142D). The BA.2 had 28 signature mutations in Spike, compared to 30 in BA.1. This was due to two revertant mutations, S446G and S496G, in the receptor-binding domain (RBD), making BA.2 somewhat similar to Wuhan-Hu-1 (WT), which had G446 and G496. The molecular dynamics simulations showed that the RBD consisting of G446/G496 was more stable than S446/S496 containing RBD. Thus, our analyses suggested that BA.2 evolved with novel mutations (i) to maintain receptor binding similar to WT, (ii) evade the antibody binding greater than BA.1, and (iii) acquire mutation of the Delta variant that may be associated with the high infectivity.

## 1. Introduction

Severe Acute Respiratory Syndrome coronavirus 2 (SARS-CoV-2), the causative agent of Coronavirus Disease 2019 (COVID-19) has been evolving, in the form of variants, since its emergence in 2019 [1]. Several variants containing different mutation clusters have emerged, comparable to the ancestral Wuhan-Hu-1 strain [2,3,4,5]. Based upon a number of infection-related criteria—including transmissibility, disease severity, decrease in neutralization, responsiveness to therapeutics, and detection sensitivity—the variants have been classified as a variant of concern (VOC), variant of interest (VOI), or variant being monitored (VBM). To date, five VOCs—Alpha (B.1.1.7), Beta (B.1.351), Gamma (P.1), Delta (B.1.617.2), and Omicron (B.1.1.529)—have been identified.

The most recently identified variant is Omicron, which includes lineage B.1.1.529 and sublineages BA.1, BA.1.1, BA.2, and BA.3, as classified by the World Health Organization (https://www.who.int/en/activities/tracking-SARS-CoV-2-variants accessed on 25 February 2022) [6,7]. A list of VOCs, their origin country and date, and significant mutations in S-protein are listed in Table 1. To gain insight into the BA.2 mutation profile, we analyzed available sequences (*n* = 8660) (as of February 2022) for the prevalence of BA.2 signature mutations. We also analyzed available structures of Spike receptor-binding domain (S-RBD) of Wuhan-Hu-1 or Omicron strains in complex with monoclonal antibodies (mAbs) to understand the function of mutations at the interface. Our analyses showed a significant difference in the number and distribution of mutations between BA.2 and Omicron BA.1. The structural data showed that BA.2 evolved such that it maintained critical contacts with ACE2 that were essential for viral entry, yet also had the capability to escape (or reduce) the binding of mAbs. A combination of these two factors rendered BA.2 an alarming variant that could impact current and future vaccination strategies.

## 2. Results

### 2.1. Prevalence of BA.2 Signature Mutations

High-quality, high-coverage sequences (*n* = 8660) from GSAID (Global Initiative on Sharing Avian Influenza Data) [8] were used to identify the distribution of mutations in BA.2. Since BA.2 was not classified as a variant in the GSAID repository, we used a combined search criterion (VOC Omicron GRA plus Spike_T376A) to filter the sequences corresponding to BA.2. In addition, we used NextClade [9] and an in-house Python script to process sequences for the identification of the BA.2 signature mutations. Overall, we identified a total of 50 signature mutations in the BA.2 variant (with ~100% prevalence), compared to 48 mutations present in Omicron BA.1 [10] (Table 1 and Table 2). Additionally, 17 mutations of BA.1 were not present in BA.2, and BA.2 had 19 novel mutations that were not signature mutations of BA.1. 

More than half (56%) of all BA.2 signature mutations (28 out of 51) were present in the S-protein (Table 2 and Table 3). In contrast, 75% of all mutations in BA.1 were present in the S-protein BA.1 [10] (Table 2). These signature BA.2 mutations within S-protein were ~100% correlated (i.e., 100% coexisting). Additionally, 8 of 28 mutations (T19I, PPA25-27Del, G142D, V231G, S371F, T376A, D405N, and R408S) were unique to BA.2 (i.e., these mutations were not present in BA.1), and 12 of 32 mutations (A67V, T95I, VYY143-145Del, N211Del, S212I, R214EPEins, S371L, G446S, G496S, T547K, N856K, and L981F) within the BA.1 S-protein were not present in BA.2. There were 20 common mutations between BA.1 and BA.2. Mutation at position 371 was present in both BA.2 and BA.1. However, BA.2 had S371F, whereas BA.1 had S371L mutation. It was interesting to note that most mutations (24 out of 28) in BA.2 S-protein were in the S1 subunit—which participates in the initial binding of the receptor, and is cleaved during virus entry. A notable mutation in BA.2 is G142D, a signature mutation in the Delta variant [3].

### 2.2. Analysis of Structural Data and Impact of Mutations on Overall S-RBD Structure

As of 25 February 2022, 571 S-protein-related structures have been deposited to the Protein Data Bank (PDB) [11]. Of these, 88 were solved by X-ray crystallography, while 483 were solved by Cryo-electron microscopy (Cryo-EM). These structures included S-protein in apo form (i.e., alone), S-protein (or S-RBD) in complex with ACE2, and S-protein (or S-RBD) bound to antibodies/nanobodies. These structures also included 23 Omicron variant S-protein structures either in apo form or in complex with ACE2 or in complex with an antibody. In total, 22 of these structures were solved by Cryo-EM with resolutions ranging between 2.45 Å and 3.88 Å, whereas one structure was solved by X-ray crystallography (PDB entry 7TN0) [12]. Comparative analyses of the S-protein apo structure with S-protein (or S-RBD), in complex with ACE2 and/or antibody, provided insights into the structural changes adopted by S-protein to bind the cofactors.

To assess if variant-specific mutations imparted any structural changes to the overall structure of S-RBD, we superposed S-RBD from Wuhan-Hu-1 S-RBD/ACE2 complex (PDB entry 6M0J) [13] on the S-RBD from Omicron BA.1 S-protein/ACE2 complex (PDB entry 7T9K) [14]. The two S-RBDs superposed exceptionally well, with a root mean square deviation (RMSD) less than 0.5 Å between 196 Cα atoms of the S-RBDs. This comparison suggested that, despite a large number of mutations (16 in total) in Omicron BA.1 S-RBD, the overall structure of S-RBD remained unchanged. The PDB entry 7TN0 [12] contained Omicron BA.1 S-RBD, bound to a broadly neutralizing sarbecovirus monoclonal antibody (mAb) S309 (the parent mAb of sotrovimab) and human ACE2. To assess if the mutations in BA.1 caused the structural changes in Omicron BA.1 S-RBD upon binding to both ACE2 and mAb, we superposed the S-RBD structure from PDB file 7TN0 (Omicron S-RBD) onto S-RBD of Wuhan-Hu-1 (PDB entry 6M0J) [13]. The S-RBDs from these strains superposed exceptionally well, with less than 0.5 Å RMSD between 196 Cα atoms, suggesting that no significant change in the overall structure of S-RBD occurred upon binding to mAb or ACE2. This structural analysis suggested that neither variant-specific mutations nor binding to mAbs caused any significant change in the structure of S-protein. Hence, the impacts of mutations on the binding of mAbs were likely through local structural changes such as readjustment of sidechains or loop conformations that could affect direct polar/nonpolar interactions between S-protein and mAbs.

### 2.3. Impact of Mutations on S-RBD/ACE2 Interaction

The structure of Omicron BA.1 bound to ACE2 (PDB entry 7T9K) [14] showed that 7 BA.1 signature mutations (G446S, T478K, E484A, Q493R, G496S, Q498R and N501Y) were within interacting distance of ACE2 [10]. Additionally, K417 and Y505 interacted with ACE2 residues (PDB entry 6M0J [13], representing a WT S-RBD (Wuhan-Hu-1). However, interactions of K417 and Y505 were lost when BA.1 acquired K417N and Y505H mutations. Two recent reports showed that Omicron BA.1 S-RBD bound with ACE2 with greater affinity than WT S-RBD [14,15]. We used the PDBePISA server [16] to determine the interactions between BA.1 S-RBD and ACE2 and WT S-RBD and ACE2 using PDB entries 7T9K and 6M0J, respectively. No significant difference in the buried surface area was noted, but there were two additional interactions in BA.1: a salt-bridge between R498 (as a result of Q498R in BA.1) and a hydrogen bond between S496 of BA.1 S-RBD and K353 of ACE2. These additional interactions could be partially responsible for a marginally better binding of BA.1 S-RBD to ACE2 than that of WT S-RBD to ACE2.

### 2.4. BA2 Unique Mutations in Relation to mAb Binding

**Figure 1:**
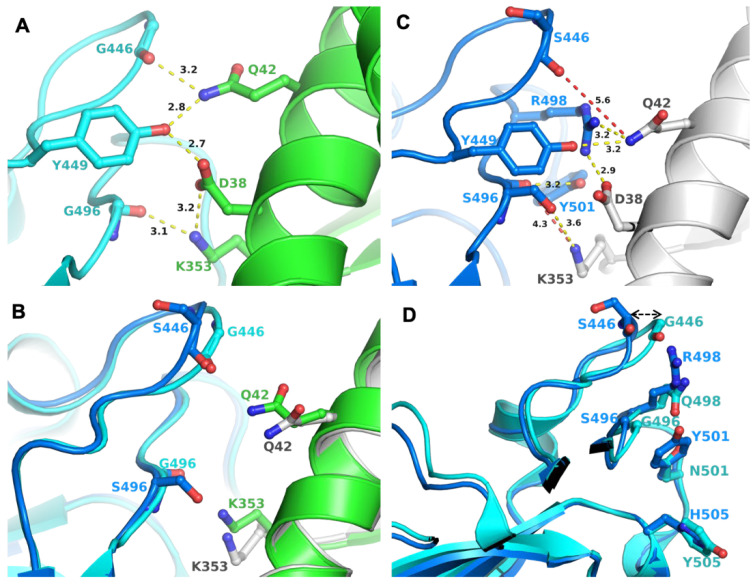
Interactions between S-RBD and ACE2 and impact of mutations. (**A**) Panel A shows the interaction network between S-RBD and ACE2, created by G446 and G449 (as in BA.2); the two residue positions that reverted to WT sequence in BA.2 compared to BA.1, which has S446 and S496, respectively. Figure generated from PDB entry 6M0J [13]. The amino acid residues are shown in the ball-and-sticks representation. The S-RBD is in cyan and ACE2, in green. The carbon atoms are rendered in the same color as the molecule (S-RBD or ACE2). In this and subsequent panels and figures, oxygen atoms are red and nitrogen atoms are blue. All distances in this and in subsequent figures are in Å. (**B**) Panel B shows the conformational change in the loops containing G/S446 and G/S496. WT-like S-RBD is in cyan and BA.1 S-RBD is in teal. ACE2 of WT S-RBD is in green and that of BA.1 is in gray. (**C**) Panel C shows that Mutation G446S and G496S (as in BA.1). These mutations cause a conformational change of the loop comprising 446 and 496 positions. Figure generated from PDB entry 7T9K, representing the BA.1 S-RBD/ACE2 complex [14]. (**D**) Panel D shows common mutations (except S446 in BA.1 and G446 in BA.2) lined at the interaction surface of S-RBD and ACE2. The color code for molecules is the same as in Panel C.

Mutation K417N was retained in BA.1, but S496 reverted to G496 (as in the WT). There were two mutations in BA.2, which reverted to the WT residues, i.e., to G446 and G496. In the crystal structure of WT S-RBD/ACE2 complex (PDB entry 6M0J), G446 and G496 participated in a network of interactions (through their backbone) that also involved Q42, D38, and K353 from ACE2 and G446, Y449 and G496 from S-RBD (Figure 1A). These interactions were expected to remain unchanged with G446S and G496S mutations (as in BA.1), as they were through the peptide backbone. However, there were two additional signature mutations in the vicinity of 446 and 496: Q498R, and N501Y. The larger side chains of R498 and Y501 required adjustments in a local structure achieved by the change in loop conformation housing G446/S446 (Figure 1B). A similar conformational change in the loop housing G496/S496 was less evident (Figure 1B). An adjustment in loop conformation rendered the loss of S446/S496 backbone interactions with ACE2, as the backbone atoms of S446 and S496 were not within interacting distance of ACE2 residues (shown in the red dotted line) (Figure 1C). However, new interactions between BA.1 S-RBD and ACE2 were generated, compensating for the loss of interactions through the backbone of S446 and S496. Conformational adjustments in this region may also have been due to the properties of glycine residue or other mutations in this region in different VOCs, including Q498R (BA.1 and BA.2), G496S (BA.1), N501Y (Alpha, Beta, Gamma, BA.1 and BA.2), and Y505H (BA.1 and BA.2) (Figure 1D). Hence, it appeared that the BA.2 variant retained the interactions of BA.1 with ACE2 and reacquired the interactions seen in the Wuhan-Hu-1.

**Figure 2:**
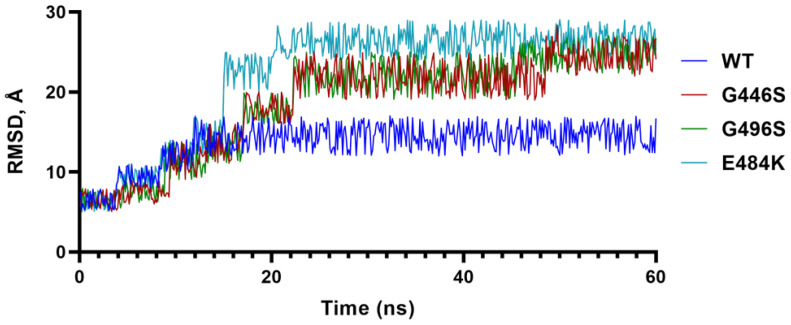
Molecular dynamics simulation. Trajectory of the root-mean-square deviation of S-RBD Cα-atoms of E484A, G446S/G496S and G446/G496 over 100 ns MD simulations.

To further understand the impact of G residues at 446 and 496 as in the WT and BA.2, we conducted molecular dynamics (MD) simulations of G and S containing S-RBDs, and included the E484A mutation as a comparison (Figure 2). The results showed that the G446/G496 combination stabilized at 15 ns, whereas S446/S496 takes longer (~23 ns) to stabilize and the RMSD of S446/S496 remained greater than G446/G496. These data suggested that the S-RBD containing G446/G496 was more stable than S446/S496, and that greater stability of G446/G496 may be one of many advantages for BA.2 to evolve with reverted WT-like residues.

**Figure 3:**
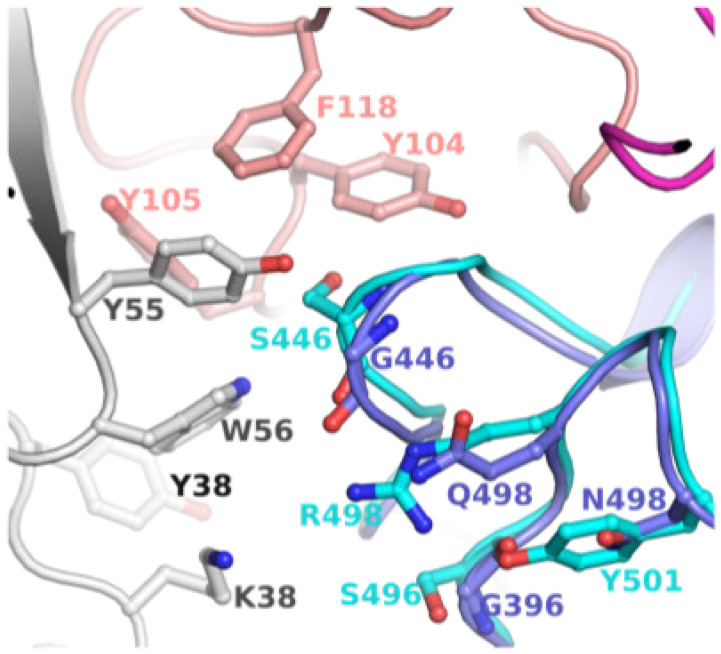
Position of G446/G449 in relation to mAb. This figure shows mAb (AZD1061) bound WT S-RBD (PDB file 7L7E). To assess the change in loop conformation housing G446 and S446, S-RBD of BA.1 bound to S304/309 mAbs (PDB entry 7TN0) is superposed. The WT S-RBD is rendered in purple ribbons, while BA.1 is in cyan ribbons. The heavy and light chains of AZD1061 are rendered in deep salmon and grey, respectively. The carbon atoms are the same color as the ribbons. The other atoms are colored as defined in Figure 1.

To explore the possibility of revertant mutations S446G and S496G evolved to evade mAb binding, we examined available structures of S-protein (or S-RBD) bound to antibodies. A crystal structure of WT S-RBD in complex with human monoclonal antibodies AZD8895 (tixagevimab) and AZD1061 (cilgavimab) was reported (PDB entry 7L7E) [17]. The combination of the two mAbs has been considered a long-acting regimen, developed by AstraZeneca and Vanderbilt [18]. The mAb bound S-RBD structure is shown in Figure 3. To assess the change in loop conformation housing G446 and S446, we superimposed the S-RBD of BA.1 bound to S304/309 mAbs (PDB entry 7TN0) [12]. This model clearly demonstrated that G446 and 496 were part of the mAb binding region which contained a cluster of hydrophobic residues. The revertant mutations (S446G and S496G), in combinations with other BA.2 in this region, could have evolved to escape the binding of AZD8895, AZD1061, S304 and/or S309.

**Figure 4:**
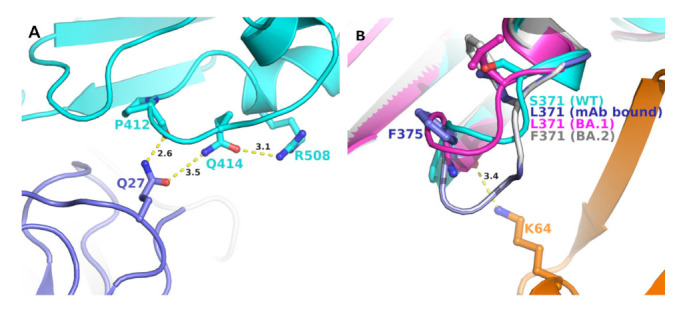
Position of unique BA.2 mutations at the interface of mAb S409 and S403 and S-RBDs. (**A**) Panel A shows the interaction network of R408 with S403 mAb light chain (purple) and S-RBD (BA.1) (cyan). A unique mutation of BA.2 (R408S) is expected to disrupt this interaction pattern and, thereby, the binding affinity of the mAb with S-RBD. (**B**) Panel B shows the change in the conformation of the loop containing S371 (WT, cyan), L371 (BA.1, magenta) (PDB entry 7T9K), BA.1 bound to S304 mAb (purple) (PDB file 7T90), and modeled S371F BA.2 mutation (grey). The interactions between mAb S409 heavy chain orange) and S-RBD (BA.1) is shown in a dotted line.

Four other mutations in BA.2 S-RBD (S371F, T376A, D405N, and R408S) differed from BA.1 (Table 2). Analyses of BA.1 S-RBD/S304/S309 crystal structure complex also provided some insight into the evolution of BA.2 to escape (or reduce) binding with these two mAbs. In BA.1 S-RBD-bound S304 crystal structure, R408 formed a hydrogen bond with Q414, which, in turn, formed a hydrogen bond with Q27 of S304 light chain (Figure 4A). Glutamine 27 of S309 also interacted with the mainchain C=O group of P412. A mutation R408S would most certainly disrupt this interaction network, leading to a reduced mAb binding to S-RBD if not altogether abolished. Residue position 371 was part of the loop formed by residues 366 to 375, a highly flexible region, as reported previously [12]. Additionally, this region already contained two phenylalanine residues. A mutation S375F (as in BA.2) (Figure 4B) added another phenylalanine, rendering an extremely hydrophobic region, which would most likely affect loop conformation, and any conformational change in this loop region is bound to impact interactions between S-RBD and heavy chain of S304 mAb.

### 2.5. BA2 Unique Mutations in Relation to N-Terminal Directed mAb Binding

The N-terminal domain of the BA.2 S1 subunit had four unique mutations (Table 2). One of these mutations, G142D was a signature mutation of the highly infectious and pathogenic Delta variant [19]. We previously showed how the G142D mutation could evade mAbs [3]. In addition to G142D, there were at least 4 more mutations that were present in Delta variant but reverted to either WT or to a different mutation in BA.2. Mutation T19R in Delta was T19I in BA.2, and T95R, L452R and D950N (all three reverted to WT) in BA.2 (Table 2). Thus, BA.2 could evade mAb recognition through a similar mechanism as the Delta variant.

### 2.6. BA2 Unique Mutations in ORFs Other than the S-Protein

In addition to mutations in BA.2 S-protein, there were 22 highly prevalent mutations (~100%) throughout the rest of the BA.2 genome. In total, 11 of these 22 mutations were unique to BA.2 (i.e., not present in BA.1). Only 5 out of the 16 mutations were unique to BA.1 (Table 3). Additionally, there were 11 common mutations between BA.1 and BA.2. Unique mutations in BA.2 in different nonstructural proteins (nsps) could have diverse roles in altering intra- and inter-nsp interactions. For example, mutation R391C in nsp14 (exoribonuclease) was close to the nsp12 (RNA dependent RNA polymerase) in the complex, representing a replication–transcription complex (RTC), consisting of nsp7, nsp8, nsp9, nsp10, nsp12, nsp13, and nsp14 (PDB entry 7EGQ) [20]. Hence, this mutation could impact the arrangement of the RTC.

Similarly, a common (BA.2 and BA.1) nsp5 (M^pro^) mutation, P132H, was located between two domains of nsp5 crystal structure bound to the Pfizer protease inhibitor PF-07321332 (PDB entry 7VH8) [21]. Mutation H132 can form a salt bridge with E240 from the adjacent domain and provide additional stabilization between the two domains. Unfortunately, extrapolating the function of mutation in nsps is not as straightforward as with mutations in S-protein mutations.

## 3. Discussion

SARS-CoV-2 has been evolving, in the form of variants and mutations in the Wuhan-Hu-1 virus that emerged more than two years ago. Most of these variants have been more infectious and pathogenic than the ancestral Wuhan-Hu-1 virus. As with all RNA viruses, the evolution of SARS-CoV-2 and the development of subsequent VOCs was not unique. However, for a virus equipped with proofreading machinery (nsp14), the rapid rate of viral evolution is surprising. In this work, we presented analyses of the mutations in BA.2 and compared them with BA.1, the original Omicron variant. It appeared that BA.2 evolved to retain a majority of the mutational profile of BA.1, but acquired additional mutations, such as G142D of Delta variant, to escape (or reduce) binding with mAbs, and reverted at some position to WT residues (such as G446 and G496) as in) to maintain the receptor binding properties of Wuhan-Hu-1. Additionally, the BA.2 variant evolved to harbor particularly relevant mutations that were present at the interface with S-protein and antibodies. The extrapolation of function for mutations in ORFs other than the S-protein encoding region was not straightforward for two reasons: (i) the number of reported S-protein structures was far more than other nsp structures, and (ii) it was difficult to structurally predict the impact of mutations in proteins that were largely enzymatic in function (such as nsp3, nsp5, nsp12 and nsp13), unless a highly conserved active site was mutated.

Regarding the mutation positions in BA.2 at the interface of S-protein and antibodies, one could wonder what factors contributed to the rapid evolution of this virus. Potentially, the virus evolved under the pressure of previously existing antibodies, either induced by vaccination (as very recently reported [22]), or by previous infections. Alternately, perhaps new lineages emerged from unvaccinated or immunocompromised individuals.

In summary, the evolution of a virus like BA.2 appeared extraordinary, as BA.2 seemingly evolved to encompass the best of both worlds: reduced susceptibility to neutralizing antibodies, yet with receptor-mediated entry activity at least as good as the wild-type virus. 

## 4. Materials and Methods

### 4.1. Sequence Acquisition and Analysis

The prevalence of each mutation in BA.1 and BA.2 was obtained from the GISAID repository [8]. These sequences were aligned using the MAFFT [23], MEGA X [24] or JalView [25] sequence alignment programs to identify BA.2 signature mutations. These sequences were analyzed for amino acid changes using Nextclade and later processed using an in-house Python script to identify the prevalence of mutations in BA.2. Any mutation with greater than 50% prevalence was considered a signature mutation.

### 4.2. Structural Analysis

An in-house R program was written to retrieve sequences from the Protein Data Bank (www.rcsb.org accessed on 25 February 2022). Specific structures were extracted using the ‘grepl’ function of the R package ‘dplyr’. The structures were then downloaded and analyzed using either the Schrodinger Suite (Schrodinger LLC, New York, NY, USA) or PyMol [26]. 

### 4.3. Molecular Dynamics (MD) Simulation

The crystal structure of S-RBD bound ACE2 (PDB file 6M0J) [13] was used as WT SARS-CoV-2 S-RBD. All MD simulations used TIP3P [27] water-filled, truncated, isometric, octahedron periodic boxes containing solutes to a 12 Å depth. A minimum of 10 Na^+^ and Cl^-^ ions were used to neutralize the charge and to increase ionic strength to 145 mM. Monovalent ion positions were randomized at 5.0 Å from other solute atoms and 3.0 Å from each other using different seeds to generate five distinct model replicates. The simulation was run at a temperature of 300 K with a pressure of 1.013 bar for 100 ns. Trajectories and trajectory files were analyzed and generated by VMD [28]. 

## 5. Limitations

All the sequences included in the analysis of mutations in BA.1 and BA.2 (*n* = 8660) were obtained from the GISAID repository [8]. These sequences were of high quality and high coverage. However, in many instances, the sequences deposited in the GISAID contained gaps in different genes, limiting the identification of mutations in some genes. Additionally, the more the sequences are reported, the more the prevalence of mutations could change by one or two percent. Nonetheless, the overall conclusion is expected to remain unchanged.

## Figures and Tables

**Table 1:** Variants of concern (VOCs), country and date of origin, and mutations in S-protein.

VOC	Country and Date of Origin	Mutations in S-Protein
Alpha	The United Kingdom, September-2020	∆H69, ∆V70, ∆Y144, E484K, N501Y, A570D, D614G, P681H, T716I, S982A, and D1118H
Beta	South Africa, May-2020	L18F, D80A, D215G, ∆L242, ∆A243, ∆L244, R246I, K417N, E484K, N501Y, D614G, and A701V
Gamma	Brazil, November-2020	L18F, T20N, P26S, D138Y, R190S, K417T, E484K, N501Y, D614G, H655Y, and T1027I
Delta	India, October-2020	T19R, V70F, T95I, G142D, DelE156-, F157-, R158G, A222V, W258L, K417N, L452R, T478K, D614G, P681R, and D950N
Omicron (BA.1)	South Africa, November-2021	A76V, T95I, Y145del, L212I, G339D, S371L, S373P, S375F, K417N N440K, G446S, S477N, T478K, E484A, Q493R, G496S, Q498R, N501Y, Y505H, T547K, D614G, H655Y, N679K, P681H, N764K, D796Y, N856K, Q954H, N969K, and L981F

**Table 2:** Unique mutations in BA.1 and BA.2, together with a mutation in WT and Delta variants within the S-protein.

BA.1 (Original)	BA.2	WT	Delta
	T19I		T19R
	PPA25-27Del		
A67V			
T95I		T95	T95I
	G142D		G142D
VYY143-145Del			
N211Del			
L212I			
	V213G		
R214EPEins			
S371L	S371F		
	T376A		
	D405N		
	R408S		
G446S		G446	
		L452	L452R
G496S		G496	
T547K			
N856K			
		D950	D950N
L981F			

**Table 3:** Unique BA.1 and BA.2 mutations in genes other than S-protein.

BA.1 (Original)	BA.2
M:D3G	
	N:S413R
	nsp1:S135R
	nsp3:T24I
nsp3:K38R	
nsp3:L1266I	
	nsp3:G489S
nsp3:A1892T	
	nsp4:L264F
	nsp4:T327I
	nsp4:L438F
nsp6:I189V	
	nsp14:R391C
	nsp15:T112I
	ORF3a:T223I
	ORF6:D61L

## Data Availability

All programs and data used in this study are available upon request.
